# Finite Element Simulation of the Machining Process of Boiling Structures in a Novel Radial Heat Sink for High-Power LEDs

**DOI:** 10.3390/ma13183958

**Published:** 2020-09-07

**Authors:** Jianhua Xiang, Zeyu Liu, Chunliang Zhang, Chao Zhou, Conggui Chen

**Affiliations:** School of Mechanical and Electrical Engineering, Guangzhou University, Guangzhou 510006, China; lc979262679@163.com (Z.L.); furongbridge@163.com (C.Z.); czhou@gzhu.edu.cn (C.Z.); cg6568@163.com (C.C.)

**Keywords:** FE simulation, radial heat sink, fabrication, microgrooves, high-power LEDs

## Abstract

A phase change heat sink has higher heat transfer efficiency compared to a traditional metal solid heat sink, and is thus more preferred for the heat dissipation of high-power light-emitting diodes (LEDs) with very high heat flux. The boiling structure at the evaporation surface is the biggest factor that affects heat sink resistance. It is necessary to investigate the plastic deformation law during the machining process of boiling structures. In this study, a novel phase change radial heat sink was developed for high-power LED heat dissipation. First, a working principle and a fabrication process for the heat sink were introduced. Subsequently, to achieve an excellent heat dissipation performance, the machining process of boiling structures was numerically simulated and investigated. To be specific, plastic deformation generated during the formation was analyzed, and key parameters related to the morphology of the boiling structures were discussed including feeding angles and machining depths. Moreover, the finite element (FE) simulation results were compared with those of experiments. Last but not least, the heat transfer performance of the fabricated heat sink was tested. Results showed that the developed heat sink was well suited for a high-power LED application.

## 1. Introduction

A light-emitting diode (LED) refers to an electroluminescent (EL) device, which is different from an incandescent lamp, a fluorescent lamp, and other traditional lighting sources in terms of the luminescence mechanism. The LED spectrum contains no infrared ray; it is thus difficult to dissipate heat through radiation. The LED chip is encapsulated in a confined and narrow space, surrounded by fluorescent powder and a lens, thereby making it difficult to dissipate heat through convection. Therefore, high-power LEDs can only dissipate heat through conduction. The area of the high-power LED chip is generally about 1 mm × 1 mm, and the input power is equal to or greater than 1 W, i.e., the heat source has a small heating area and a high heat flow density. In the case of a metal solid heat sink, the volume and the surface area of the aluminum or copper heat sink are restricted due to the narrow encapsulation space. An increase in the cooling area leads to an increase in the heat transmission distance, resulting in a sharp decrease in cooling efficiency. To address high heat flow density under the condition of narrow space with regard to the LED’s thermal control problem, more efficient cooling patterns must be used to replace the conventional cooling techniques.

Over time, phase change heat transfer technology has become the focus for the future development direction of high-power LEDs. Many researchers have contributed to the development of the theory, method, design, and manufacture of the phase change heat sink [1,2,3,4,5,6,7,8]. A phase change heat sink can dissipate the heat from a high-power LED’s heat source in real time. The external heat load is first introduced into boiling structures at the evaporation surface. A liquid working medium inside the structure then vaporizes under the influence of the heat. The steam flows rapidly and fills the steam chamber. When the steam makes contact with the condensation surface, it coagulates rapidly and releases latent heat of vaporization. Subsequently, the liquid working medium at the condensation surface returns to the evaporation surface through capillary force by the capillary core. When the cycle repeats in this way, the heat can be transmitted from the evaporation surface to the condensation surface continuously. It should be noted that the boiling efficiency of the evaporation surface is the biggest factor that affects heat sink resistance [9]. During the formation of boiling structures at the evaporation surface, ploughing–extrusion and stamping process are accompanied by severe plastic deformation in terms of nonlinear stress–strain relations and nonlinear strain–displacement relationships. It is necessary to understand its deformation law under plastic deformation theory for analysis.

With the development of modern computer technology and the connection made between plastic forming technology and the computer, the finite element method (FEM), as a type of effective numerical calculation method, has been widely used in metal plastic forming process analysis. At the same time, some commercial finite element software (ANSYS, ABAQUS, DEFORM, etc.) have become a powerful tool for the analysis of cutting processes. In the 1970s, Klamecki first introduced the finite element method (FEM) to the numerical simulation of machining [10]. Since then, the FEM has been widely used in cutting analysis, and many scholars have performed in-depth research. Representatively, Zhou and Wierzbicki presented a tension zone model of the blanking and tearing of ductile metal plates [11]. Using the tension model as a representative element, they then proposed the blanking model with a moving fracture front to predict the shear force, the plastic work, as well as the shape and size of the plastic shear zone. Breitling et al. described the state of the art in shearing equipment and summarized the results of simulating the shearing process using a modified 2D finite element method (FEM) code [12]. It was shown that the influence of process variables such as blade clearance, material properties, and tooling configurations could be simulated with a good correlation to experimental results. Hambli et al. applied a neural network for optimum clearance prediction in sheet metal blanking processes [13]. The numerical results obtained by finite element computation including damage and fracture modeling were utilized to train the developed simulation environment based on backpropagation neural network modeling. Kim et al. performed molecular dynamics simulations of plastic material deformation in machining with a round cutting edge [14]. A key observation was the marked variation in the geometry of the observed characteristic features, the most notable being the rotation and growth of the stable built-up edge as the uncut chip thickness increased. S. Atlati developed a new method on the basis of contact condition variation at the tool–work material interface to predict the built-up edge formation through finite element simulation [15]. Oliaei and Karpat investigated the effect of friction conditions on FE simulation of micro-scale machining with the presence of a built-up edge [16]. Zang investigated the effect of thermal conductivity on the degree of chip segmentation and the adiabatic shear localization using ABAQUS/Explicit [17]. They found that as thermal conductivity increases, the degree of adiabatic shear decreases. In addition, they also observed cracks located in the adjacent segments of chips.

In this study, a novel phase change radial heat sink was designed and fabricated to deal with LED heat dissipation. The operating principle of the heat sink and a relevant manufacture method were first introduced. Subsequently, the processing technology of the phase change heat sink’s boiling structures was numerically simulated and investigated. In this regard, plastic deformation generated during the machining process was analyzed, and key parameters were discussed including feeding angles and machining depths. To demonstrate the effectiveness of the simulation, the FE results were compared with those of experiments. Finally, the heat transfer performance of the fabricated phase change radial heat sink was tested.

## 2. Operating Principle and Fabrication Process of the Developed Phase Change Heat Sink

**Figure 1:**
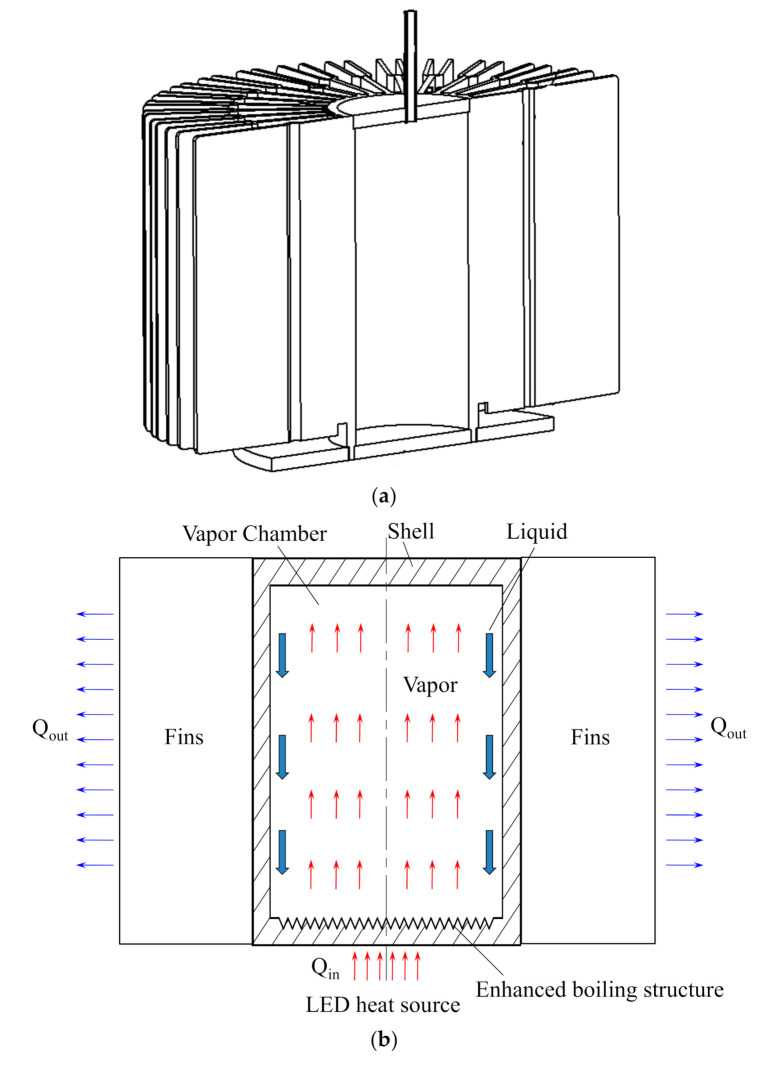
(**a**) Geometry of the developed radial heat sink for high-power light-emitting diode (LED) heat dissipation and (**b**) its operating principle.

A novel radial phase change heat sink was developed to satisfy the heat dissipation of a 200 W LED lamp. The geometry of the developed heat sink is shown in Figure 1a. It can be seen that the heat sink mainly includes two parts, i.e., a vapor chamber and fins. The heat from the LED chips was first transmitted to the wall of the vapor chamber, and then dissipated to the ambient environment through fins. The operating principle of the radial heat sink is shown in Figure 1b. The LED heat load is first introduced into the boiling structures through the evaporation surface (hot side). Subsequently, the liquid working fluid inside the chamber evaporates after absorbing the heat from the hot side. Much steam is generated quickly and fills the entire chamber. When the steam is in contact with the condensation surface (cold side), it condensates rapidly and releases latent heat. Then, the liquid working medium returns to the evaporation surface by gravity. Upon repeated cycles, the heat from the LED chips can be transmitted to the cold side and dissipated continuously to the ambient environment.

**Figure 2:**
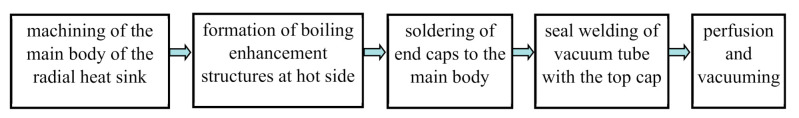
Fabrication process of the radial phase change heat sink.

The fabrication process of the radial phase change heat sink is shown in Figure 2. First, the main body of the radial heat sink was machined by tensile extrusion. Then, boiling enhancement structures at the hot side were machined through ploughing–extrusion and stamping. Subsequently, end caps (including top cap and bottom cap) were soldered to the main body of the heat sink. Afterward, seal welding was performed between the vacuum tube and the top cap. Finally, perfusion and vacuuming were conducted, and the heat sink was painted with coatings to increase heat radiation.

## 3. Finite Element Simulation

According to previous research [9], boiling convection resistance at the evaporation surface dominates in total thermal resistance. In order to minimize total thermal resistance, it is important to reduce the thermal resistance of the boiling convection, i.e., to improve the boiling efficiency of the inner surface of the hot side. Therefore, the manufacture of boiling enhancement structures at the hot side becomes the key to the radial phase change heat sink’s heat transfer performance. In this study, to improve the boiling efficiency of the boiling structures, the machining process was simulated using DEFORM-3D v11.0 to fully understand the plastic deformation law and the formation mechanism.

**Figure 3:**
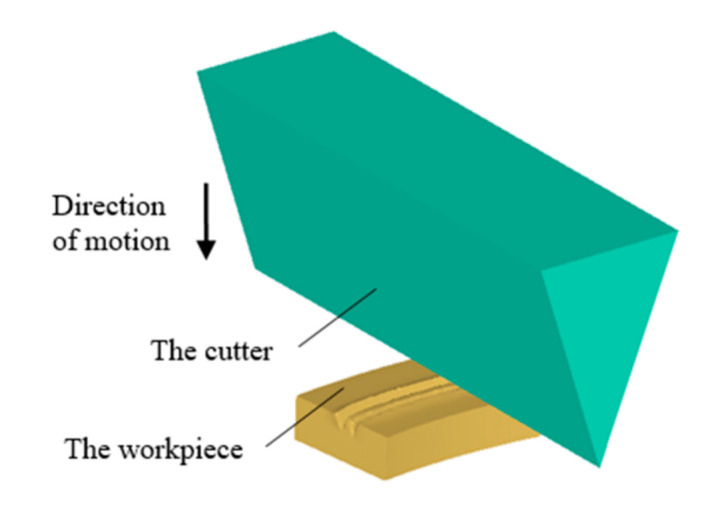
Geometry model of the workpiece and the cutting tool in simulation.

During the simulation, for the convenience of the software setting, the transformation was carried out according to the relative velocity principle, i.e., the workpiece was deemed as a fixed piece, while the cutting tool was rotating at a certain speed centered at the workpiece center. The process of machining two adjacent grooves is regarded as two different forming processes. Because of the axisymmetric characteristics of the geometry, 1/8 of the workpiece was selected for simulation, in order to reduce the amount of calculation and at the same time improve the analysis precision. The geometry model of the workpiece and the cutting tool is shown in Figure 3.

**Figure 4:**
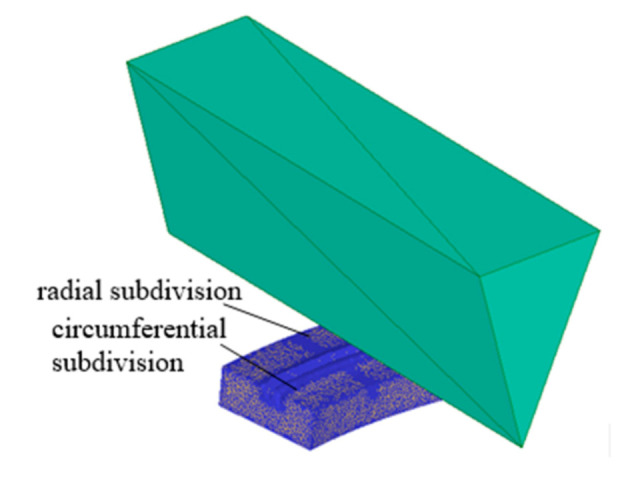
Finite element (FE) mesh model of the workpiece.

During the formation of boiling structures, plastic deformation is generated to a greater degree than elastic deformation. Accordingly, a rigid-plastic model was used during simulation, i.e., the workpiece is rigid-plastic and the tool is rigid. The workpiece was divided into 100,000 tetrahedron elements. Because of the large plastic deformation of the workpiece generated during the machining process, the meshes between the cutting tool and the workpiece were subdivided, as is shown in Figure 4. During the simulation, stamping depths were set from 0.1 to 0.5 mm, and rotation angles were set from 1° to 3°.

**Figure 5:**
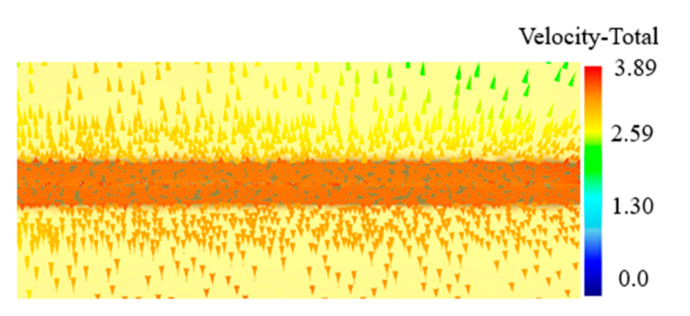
Flow vector of the metal during the stamping process.

As is indicated in Figure 5, when the cutting tool is in contact with the workpiece, the metal by the cutter starts to flow to both sides under the action of extrusion by the wedge knife. Due to the effect of the resistance of the metal from the distance, the metal flows to the unconstrained upper surface that has least resistance. With the increase in the stamping depth, increasingly more metal lifts up and a fin is formed on both sides of the cutting tool. The flow direction of the metal is shown in Figure 5.

**Figure 6:**
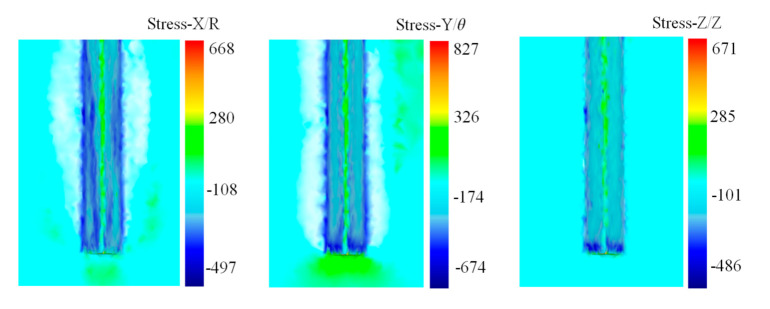
Tri-axial stress distribution during the stamping process.

From the perspective of stress distribution, under the extrusion of the stamping tool, tri-axial stresses at both walls of the groove are compressive. The largest compressive stress occurs at the edge of the groove. Tensile stress occurs at the bottom of the groove, because the metal is separated and flows to both sides. While in the non-contact zone, the stress is compressive under the extrusion of adjacent metal. Tri-axial stress distribution during the stamping process is shown in Figure 6.

## 4. Results and Discussion

### 4.1. Effect of Different Stamping Depths on the Morphology of Microgrooves

Concerning the process of stamping forming, stamping depth *a*_c_ is a crucial parameter. The stamping depth was set as 0.1 mm, 0.2 mm, 0.3 mm, and 0.5 mm during the simulation.

#### 4.1.1. Morphology of Microgrooves under Different Stamping Depths

**Figure 7:**
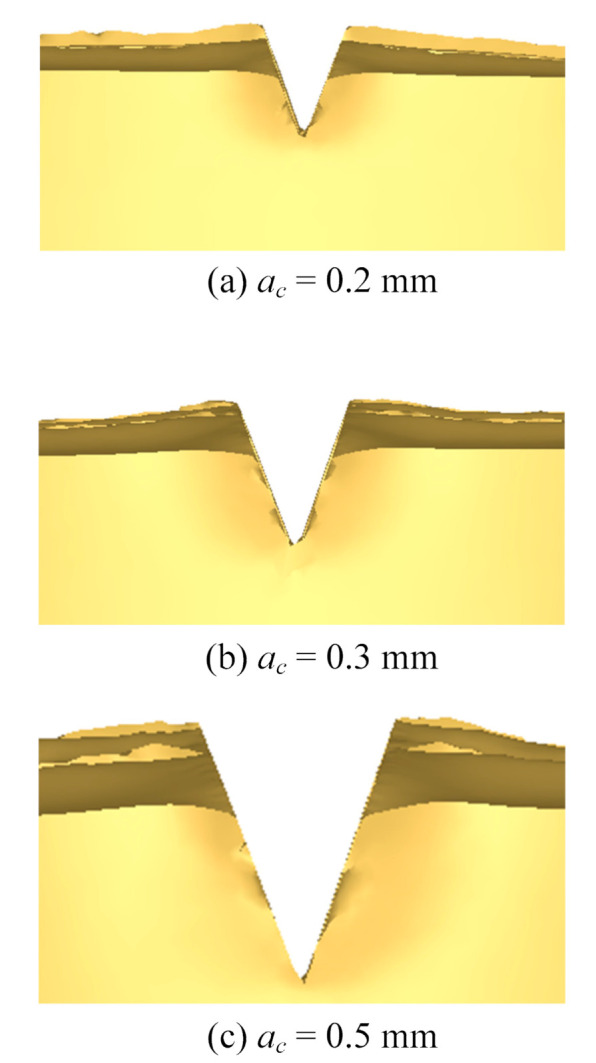
Morphology of microgrooves under different stamping depths in simulation. (**a**) *a*_c_ = 0.2 mm; (**b**) *a*_c_ = 0.3 mm; (**c**) *a*_c_ = 0.5 mm.

**Figure 8:**
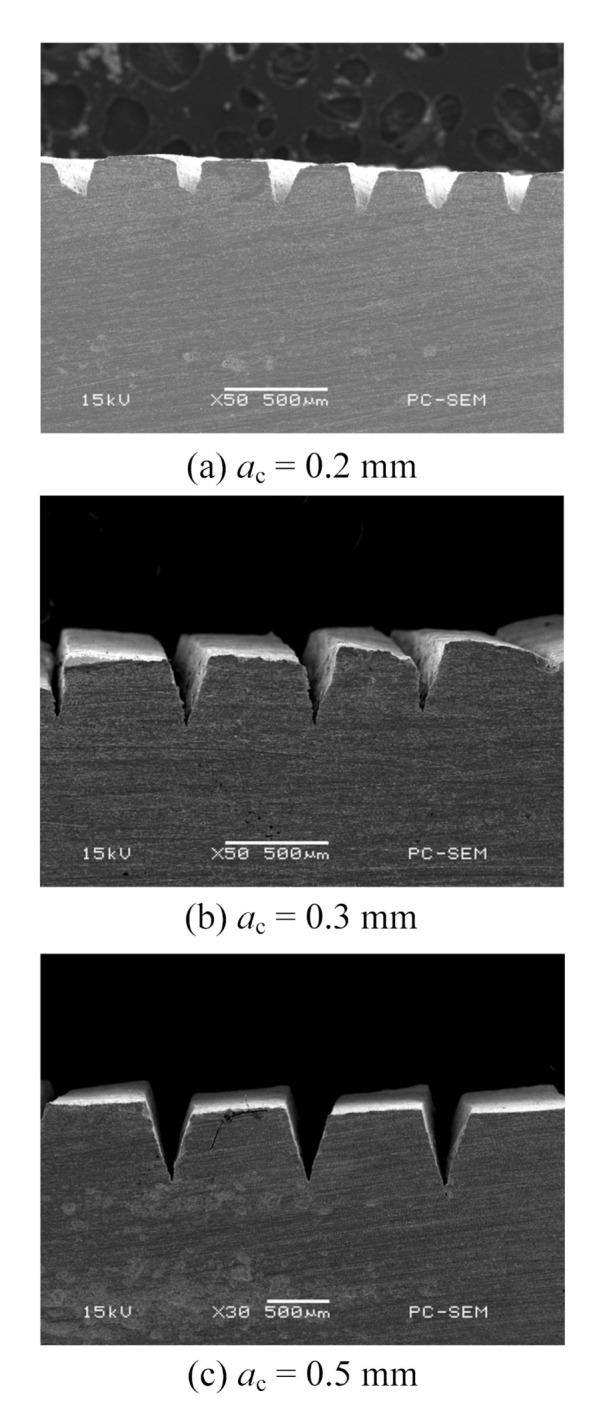
Morphology of microgrooves under different stamping depths from experiments. (**a**) *a*_c_ = 0.2 mm; (**b**) *a*_c_ = 0.3 mm; (**c**) *a*_c_ = 0.5 mm.

The morphology of microgrooves under different stamping depths in simulation is shown in Figure 7a–c. It can be seen that the V groove sections are similar under different stamping depths. With the increase in the punching depth, the depth and width of the V grooves increase as well. This occurs because the volume and size of the metal deformation area increase as the stamping depth increases. More metal flows to the free surface to form fin structures under the extrusion of the cutting tool. In addition, the height of the fin increases as the stamping depth increases. To make a comparison, the morphology of microgrooves under different stamping depths from experiments is shown in Figure 8. It can be seen that the morphology from the simulation coincides well with that from the experiments.

#### 4.1.2. Analysis of Cutting Force

**Figure 9:**
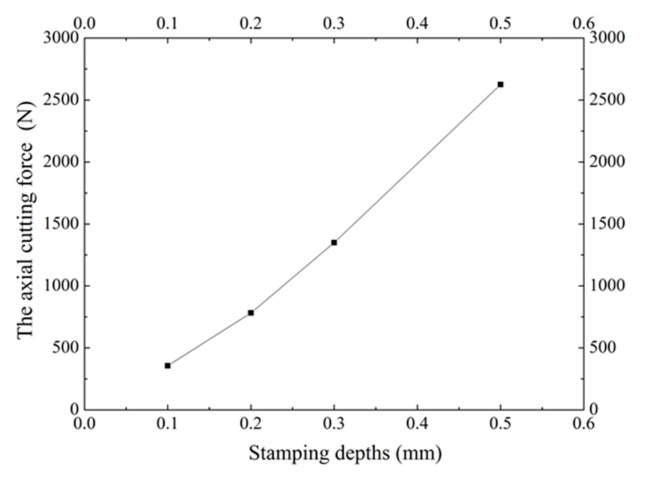
Axial cutting force under different stamping depths.

During the process of stamping, the cutting force mainly acts in the Z direction, while the radial and circumferential forces are small. Accordingly, the radial and circumferential forces were ignored in the analysis. A certain processing action is required for the cutting tool to reach the predetermined stamping depth while punching microgrooves. The simulated cutting forces in the Z direction under different stamping depths are shown in Figure 9. It can be seen that the axial cutting force (Z direction) increases as the punching depths increase. When the stamping depth is 0.1 mm, the axial cutting force is about 350 N. The axial cutting force increases relatively slowly when the stamping depth is between 0.1 mm and 0.3 mm, where the cutting force is smaller than 1500 N. When the stamping depth further increases, the volume of the flowing materials increases as well. Due to the deformation resistance of the material, the axial cutting force increases rapidly. When the stamping depth increases to 0.5 mm, the axial cutting force reaches around 2500 N.

#### 4.1.3. Stress Distribution

**Figure 10:**
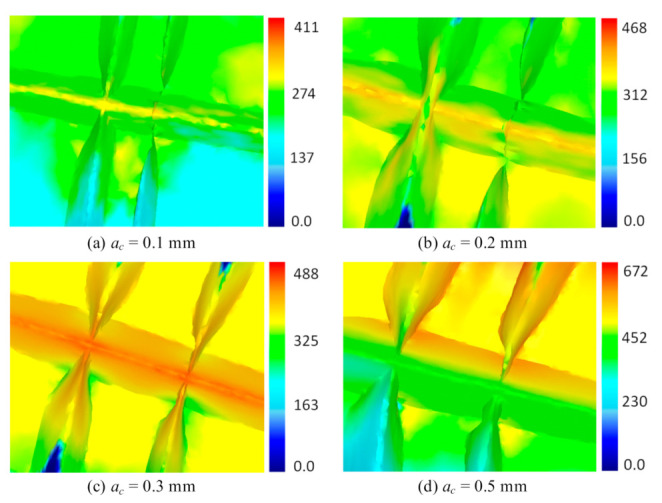
Equivalent stress distribution with different stamping depths. (**a**) *a*_c_ = 0.1 mm; (**b**) *a*_c_ = 0.2 mm; (**c**) *a*_c_ = 0.3 mm; (**d**) *a*_c_ = 0.5 mm.

Equivalent stress distribution at the microgroove with different stamping depths is shown in Figure 10. As is seen from the figure, the area of the deformation zone at the groove increases gradually as the stamping depth increases. From the perspective of stress distribution, the stress increases gradually as the punching depth increases. It is noted that the equivalent stress distribution is symmetrical to the groove. A smaller stress occurs away from the cutting tool, while a larger stress occurs at the bottom of the groove in contact with the cutter. When *a*_c_ is 0.5 mm, the stress reaches a maximum value of 672 MPa.

### 4.2. Effect of Feeding Angles on the Morphology of Microgrooves

The morphology of microgrooves is not only affected by stamping depths, but is also influenced by the feeding angle *θ*_c_. Numerical simulation was performed with different feeding angles at a certain stamping depth.

#### 4.2.1. Analysis of Plastic Forming at Different Feeding Angles

**Figure 11:**
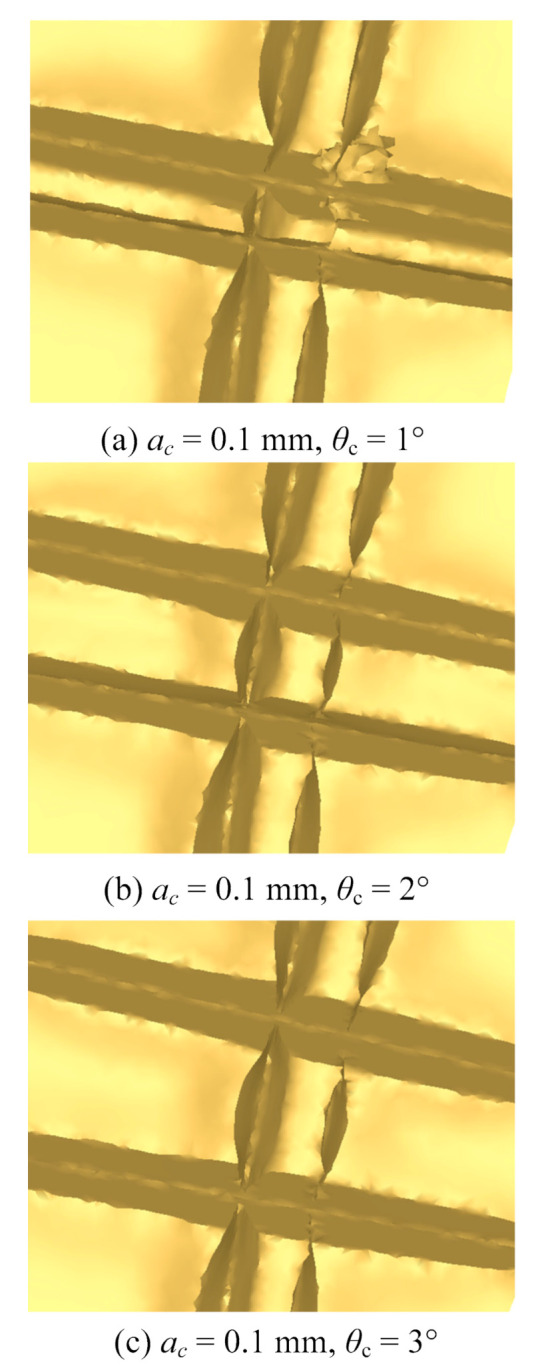
Morphology of microgrooves with different feeding angles in simulation. (**a**) *θ*_c_ = 1°; (**b**) *θ*_c_ = 2°; (**c**) *θ*_c_ = 3°.

During the process of stamping forming, a radial structure is formed due to the overlap of the radial groove with the center of the evaporation end for the phase change heat sink. The morphology of the radial structure is determined by the feeding angle, as is shown in Figure 11. A small part of the workpiece is taken out for simulation at different feeding angles because of the limit of calculation time. After extrusion from the stamping tool, the width of the slot top of the circumferential groove decreases due to metal flow. The two walls of the circumferential groove draw close to the center. Consequently, hybrid structures with grooves, holes, and fins are formed.

**Figure 12:**
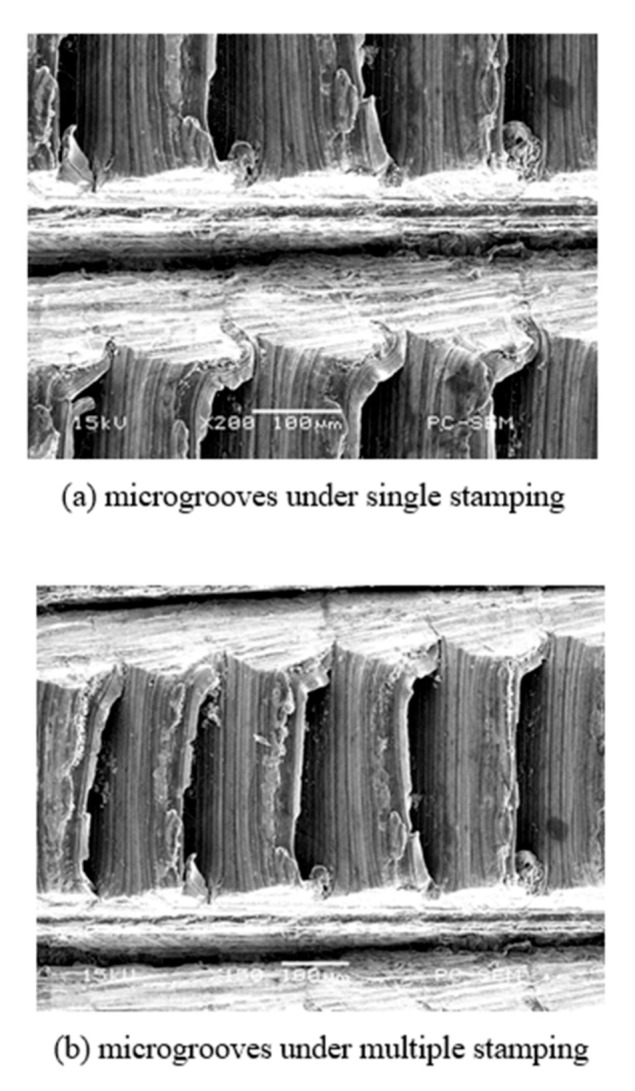
SEM section images of microgrooves under the stamping process, (**a**) single stamping; (**b**) multiple stamping.

The morphology of microgrooves under stamping on the basis of circumferential grooves machined by ploughing–extrusion is shown in Figure 12. It can be seen that a circumferential fin is first cut open during stamping, and then moves along the wedge surface of the cutter under the extrusion of the cutting tool. Metal that makes contact with the tool moves towards the circumferential grooves. Hybrid structures including microgrooves, holes, and fins are finally formed. These structures are consistent with those seen in Figure 11 in simulation.

#### 4.2.2. Analysis of Cutting Force

**Figure 13:**
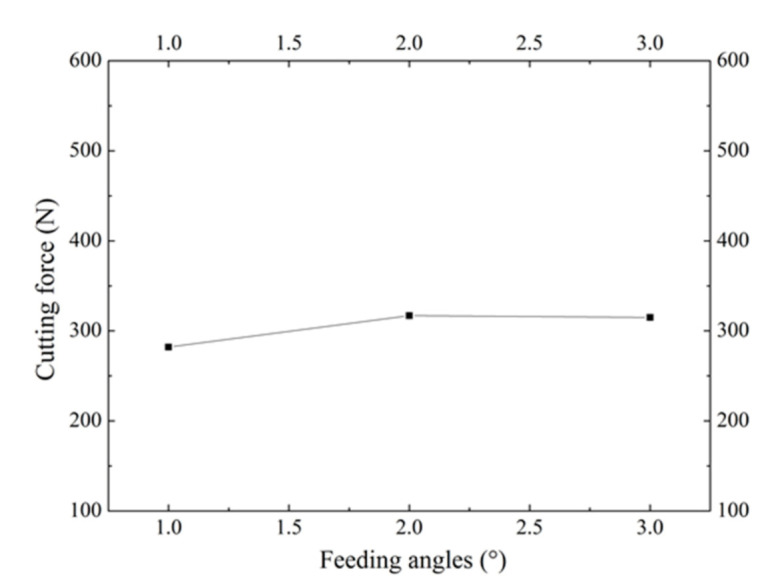
Cutting force under different feeding angles.

The axial cutting force (Z direction) with different feeding angles is shown in Figure 13. It can be seen from the figure that the curve changes very slowly, i.e., the impact of the feeding angle onto the cutting force is insignificant. When the feeding angle is small, the distance to the previous V groove is small, leading to a small thickness of metal between the two V grooves. Accordingly, it is easy for the material to deform during stamping, resulting in an axial cutting force of 300 N with a feeding angle of 1°. When the feeding angle increases, the metal thickness between the two V grooves increases as well, where the axial cutting force is similar to the single V groove forming, having an axial cutting force slightly greater than 300 N when the feeding angle is between 2° and 3°. Therefore, the main factor that affects axial cutting force is the stamping depth, whereas the feeding angle has less effect. If the feeding angle is too small, the interference part between the radial grooves has a negative effect on the circumferential microgrooves. If the feeding angle is too large, the number of radial grooves decreases, having a negative effect on the radial flow of the working medium. Taking all factors into account, the feeding angle of 2° is the most appropriate.

#### 4.2.3. Stress Distribution

**Figure 14:**
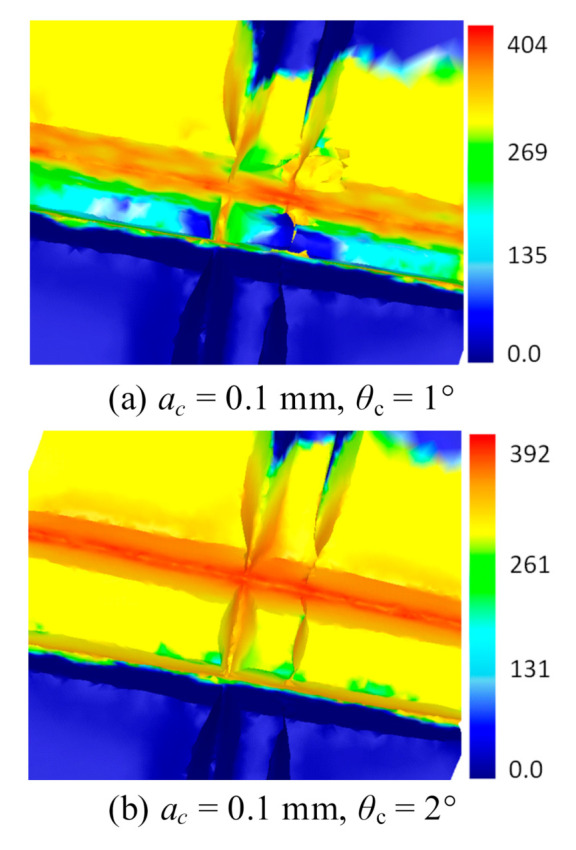

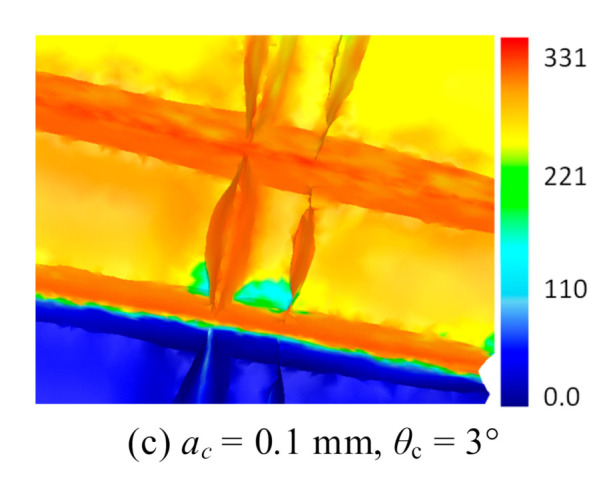
Equivalent stress distribution under different feeding angles. (**a**) *θ*_c_ = 1°; (**b**) *θ*_c_ = 2°; (**c**) *θ*_c_ = 3°.

Equivalent stress distribution at the microgroove with different feeding angles is shown in Figure 14. It can be seen that asymmetric stress distribution is formed since the previous groove cuts off the stress in the feeding direction of the tool. When the feeding angle is small, the thickness of the two grooves is small, making the central metal with a small thickness tilt due to the extrusion by the stamping tool. Thus, the stress mainly concentrates in the feeding direction of the cutter. The stress distribution at a feeding angle of 1° is shown in Figure 14a. When the feeding angle is larger, the thickness of the two grooves increases. Metal at the center produces a large deformation under the extrusion of the cutting tool, leading to a larger flow resistance. Therefore, the stress distributes on both sides of the cutting tool, as is shown in Figure 14b with a feeding angle of 2°. With the further increase in the feeding angle, the stress distribution is similar to the case under single punching, as is shown in Figure 14c with a feeding angle of 3°.

## 5. Heat Transfer Performance Testing

**Figure 15:**
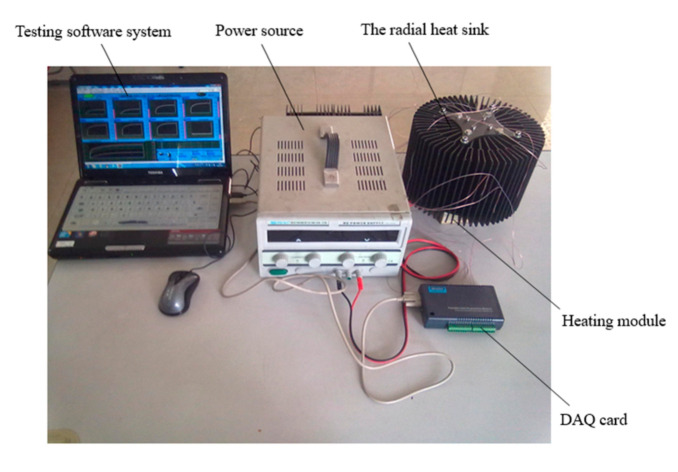
The developed heat transfer performance testing platform.

In this study, the heat transfer performance of the developed radial heat sink was tested under natural convection using the developed heat transfer performance testing platform, as is shown in Figure 15. The testing platform mainly includes a testing software system, a power source, a DAQ card, a heating module, and the radial heat sink. The testing software system was developed using LabVIEW 8.20 (National Instruments, Austin, TX, USA), to accomplish data acquisition, treatment, display, and storage. The heating module was made up of four heating rods, and every rod had a maximum output power of 100 W, to simulate the heat generated by high-power LEDs. Omega T-type copper–nickel thermocouples (TT-T-30-SLE) were applied to measure the temperature of typical points of the heat sink including the hot side and the cold side. An Advantech USB-4718 DAQ card (Advantech Co., Ltd, Taipei, China) was applied to collect the data from the thermocouples and transmit the data to the computer. In addition, a non-contact infrared thermometer was applied to check temperature error during the testing.

**Figure 16:**
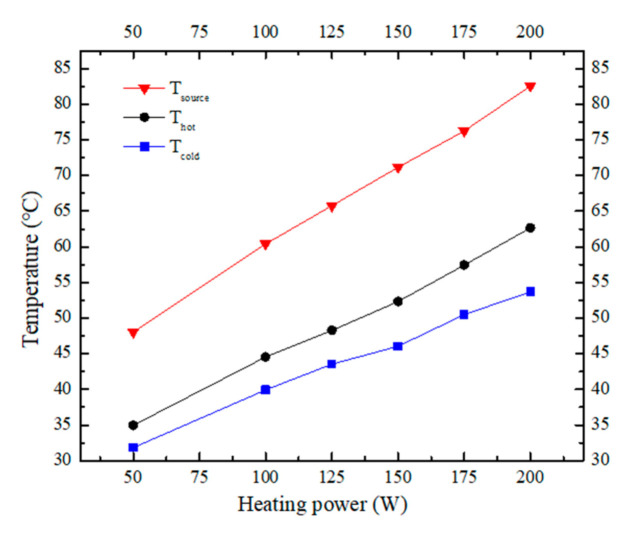
Temperature variation under different heating powers.

The temperature variation curves under different heating powers (from 50 W to 200 W) are shown in Figure 16. In the figure, T_source_ represents the temperature at the center of the heating source, T_hot_ represents the average temperature of the hot side of the heat sink, and T_cold_ represents the average temperature of the cold side of the heat sink. It can be seen that the temperature variation trends of these three curves are almost the same, i.e., temperature increases approximately linearly with the increase in the heating power. It should be noted that the maximum temperature at the center of the heat source is lower than 85 °C under the heating power of 200 W. Consequently, the developed phase change radial heat sink is well suited for high-power LED heat dissipation applications.

## 6. Conclusions

(1) This paper proposed a novel phase change radial heat sink for high-power LED heat dissipation. The working principle and the fabrication process of the heat sink were introduced.
(2) The machining process of the boiling structures was simulated using DEFORM-3D v11.0. Plastic deformation generated during the formation was analyzed. Key parameters related to the morphology of the boiling structures were discussed including feeding angles and machining depths. When the stamping depth changes from 0.1 mm to 0.5 mm, the axial cutting force increases from 350 N to 2500 N. The maximum equivalent stress can reach around 672 MPa. Considering the interference effect and the number of radial grooves, the optimal feeding angle is 2°.
(3) The FE simulation results were compared with those of experiments. Results show that they coincide well with each other.
